# Race and Racism as Structural Determinants for Emergency and Recovery Response in the Aftermath of Hurricanes Irma and Maria in Puerto Rico

**DOI:** 10.1089/heq.2019.0103

**Published:** 2020-05-22

**Authors:** Carlos E. Rodriguez-Díaz, Charlotte Lewellen-Williams

**Affiliations:** ^1^ School of Public Health, University of Puerto Rico-Medical Sciences Campus, San Juan, Puerto Rico.; ^2^ Milken Institute School of Public Health, The George Washington University, Washington, District of Columbia, USA.; ^3^ Center for Community Philanthropy, Clinton School of Public Service, University of Arkansas, Little Rock, Arkansas, USA.

**Keywords:** race, natural disaster, Puerto Rico, social determinants of health, racism

## Abstract

**Purpose:** To explore the role of race and racism in emergency response and recovery in the aftermath of hurricanes in Puerto Rico (PR).

**Methods:** Sixteen semistructured qualitative interviews were conducted between March and April 2018 with community members who had an active role in the process of response and relief efforts. Among participants, eight were from PR, and eight were of Puerto Rican descent living in the continental United States. Narrative text from interviews was analyzed using grounded theory approach and narrative analysis techniques.

**Results:** Participants were adult men and women from different municipalities in PR and diverse regions of the continental United States and with diverse professional and economic backgrounds. In the analysis of the interviews, “fitting the box” of race, race in emergency and recovery response, and community philanthropy emerged across narratives as frequent and as illustrative of the issues of race/racism and response to natural disasters in PR. Participants shared a perception that the combination of disasters, including natural disasters and historic political mismanagement, is the cause of the precarious conditions in PR in the aftermath of the hurricanes. Race was perceived as a problematic construct in the understanding of Puerto Rican identities. Racism was contextualized as part of the complicated relationship between PR and the United States and as an obstacle for adequate emergency response.

**Conclusions:** Systemic racism was perceived as a barrier to emergency and recovery response in the aftermath of natural disasters. Structural changes are required to reduce vulnerability and health inequities in PR.

## Introduction

On September 20, 2017, the history of Puerto Rico (PR) changed dramatically. Despite being in the “hurricane alley,” where the occurrence of certain natural disasters can be predicted, PR was not ready for the impact of two consecutive major hurricanes. Hurricane Irma passed by PR on September 6, and Hurricane Maria crossed the island on September 20, but the socioeconomic and structural factors that weakened local resources have been present for decades.^1^

PR is undergoing a profound and complex socioeconomic crisis,^2–4^ which includes a massive government debt that has led to unpopular austerity measures^5^ and significant emigration.^6,7^ The destruction of PR's infrastructure caused by these natural disasters has compounded these pre-existing adverse socioeconomic and political conditions.^8–10^ After nearly a year after the impact of Hurricane Maria, many were still without electricity, and the public health infrastructure was weakening.^11–13^

PR is a colony. As defined by the Constitution of the United States of America, PR is an organized but unincorporated territory of the United States since 1898 and lack self-determination. Puerto Ricans on the islands do not have full representation in US Congress and cannot vote for president. Moreover, US federal mandates take precedence over local legislation and policies in all areas of governance. The sociopolitical relationship of the United States and PR is complex, and certainly, the natural disasters brought by hurricanes Irma and Maria exposed the colonial laws and practices that limit the scope of actions that the PR has in response to emergencies.

PR contributes to the annual appropriation of funding to the Federal Emergency Management Administration (FEMA) and relies on FEMA's response in case of emergencies. However, in the aftermath of the hurricanes, FEMA's response was limited. The agency argued that it was difficult to respond to multiple simultaneous federal disaster zones in California, Florida, PR, and Texas. But based on an analysis of FEMA's plan for disaster, it was evidenced that the federal government significantly underestimated the potential damage to PR from Hurricane Maria and relied too heavily on local official and private-sector entities to handle the emergency and recovery process.^14^

Similarly, the Merchant Marine Act of 1920, known as the Jones Act, establishes that US agencies control the maritime waters and ports of PR. This Act epitomizes PR's colonial condition by restraining the ability of non-US vessels and crews to engage in trade with PR. As a consequence, a significant portion of resources for relief that could have been provided by the international communities to PR were prevented or significantly delayed. Furthermore, it has been estimated that within 5 months after the impact of Hurricane Maria, the US Department of Homeland Security had accumulated more than half a billion dollars in posthurricane expenditures in PR. However, less than a fifth of all these resources were in the hands of Puerto Rican contractors.^15^

Although PR's crisis is not generally seen as a racial matter, as argued by Negrón-Muntaner,^16^ it should be. Since the beginning, the basis to obtain colonies after the Spanish–American War of 1898 rested on the notion of white racial superiority. Likewise, the entire legal structure that defines PR as an unincorporated territory that belongs to but is not part of the United States is unapologetically racist.^16^

Discourses of racial democracy discourses of racial democracy have represented Puerto Ricans as the harmonious product of the mixture between Spaniards, “Indians” (Amerindians), and Africans. Advocates of *blanqueamiento* (whitening) also claim that Puerto Ricans have “evolved” by shedding most of their African extraction through race-mixing practices. Hence, whereas in the United States anyone with African ancestry is classified as “Black,” in PR race mixture is predominantly understood as a whitening mechanism.^17^ According to the 2010 Census, 42% of Puerto Ricans who live in the states self-identified as white, as compared with 80.5% of those living in PR. This evidences the locally specific nature of this definition of Puerto Rican whiteness. Experts from PR have argued that Puerto Ricans' beliefs about race and racism also reveal the pitfalls of a long-standing colonial dilemma between the United States and PR that makes it difficult to document structural racism.^17^

In public health research it has been argued that racism can occur in multiple levels, including internalized (i.e., incorporation of racist attitudes, beliefs or ideologies into one's worldview), interpersonal (i.e., interactions between individuals), and structural level (i.e., racist control of and access to labor, material, and symbolic resources within a society).^18^ There is significant evidence of race-associated differences in health outcomes, predominately in the United States context.^19^ However, most of this research has focused on the impact of interpersonal and individual-level racism, and less work has been conducted to document the role of race/racism at the structural level.

Similarly, except for few published studies,^20–24^ there is limited research on the role of racism in emergency and relief response in the aftermath of natural disasters. In this study, we focused on the analysis of racism at the structural level to explore the role of race in emergency response and recovery in the aftermath of hurricanes in PR from the voices of residents of PR and Puerto Ricans in the diaspora. This study was a collaboration designed to explore issues of race and racism in emergency response after natural disasters while challenging the broader philanthropic, academic, and nonprofit sectors to reignite efforts aimed at advancing racial equity.

## Methods

For this study, we conducted 16 semistructured interviews. The purpose of these interviews was to explore the role of race and racism in emergency response and recovery in the aftermath of hurricanes in PR. We used semistructured interviews, including predetermined set of open questions that also provided the opportunity for the interviewer to further explore particular themes or responses.

Participants were selected using a theoretical approach,^25,26^ which provided to recruit participants and collect, code, and analyze data simultaneously. This approach is consistent with Grounded Theory and provided to capture a diverse range of experiences, characteristics, and different levels of influence associated with individual and community preparedness and response before and in the aftermath of the hurricanes in PR. Among participants, eight were from PR and eight were of Puerto Rican descent living in the United States, defined as Puerto Rican diaspora. The inclusion of experiences from individuals from PR and in the diaspora was purposive to address the documented difference in the understanding of race and to include diverse perspectives in experiencing the impact of the hurricanes.^27^

Participants were recruited by the research team based on their active role in responding to the emergency and the aftermath of the natural disasters in PR. The research team used social media, news articles, and community networks to identify and recruit research participants. The selection of these participants followed the criteria for data saturation^28^ and information power,^27^ common to the qualitative analysis planned, and consistent with the practice of collecting information relevant to the study until data shared are repetitive or follow a pattern. All participants were adults (>21 years), able to communicate in Spanish or English, and provided verbal consent to audio record the interviews.

Participants from PR had to be residents of PR, having been in PR during and after the impact of hurricanes Irma and Maria, and having had a role in the process of emergency response and relief efforts at the community, governmental, or civil society level. The rest of the participants were people of Puerto Rican descent living elsewhere in the United States for at least 6 months before the impact of the hurricanes in PR. These participants could not have been in PR during the impact of the hurricanes and had a role in the process of emergency response and relief efforts from the diaspora (i.e., organized community relief efforts such as fundraising or organized donations of goods to be sent to PR).

All participants were recruited by a bilingual (Spanish and English) researcher who also conducted all the interviews. Face-to-face and phone interviews were digitally recorded using a phone-based software. Study procedures were approved by the Institutional Review Board of the University of Arkansas for Medical Sciences.

### Analysis

Audio recordings from the interviews were transcribed verbatim and identifying information was removed prior to analysis. The narrative text was analyzed using grounded theory approach and narrative analysis techniques.^26^ The software NVivo V.12 (QSR International) was used to facilitate the organization of the narrative data set, including interview transcripts and interview notes. After an initial review of the narrative data, the investigators jointly developed coding schemes that supported subsequent and more advanced analytic techniques. We approached qualitative data analysis as an iterative process that began as soon as data collection started and reanalysis or recoding continued throughout the project. For example, as transcripts were prepared, they were classified to identify patterns and relationships related to the research domains. Codes were assigned to the text on several different hierarchical levels and for several different content areas simultaneously.

As anticipated, several themes emerged, varying by individual and yet sharing certain themes across subject narratives. Thematic analysis was used to describe the complexities associated with race/racism and the preparation for the impact of the hurricanes and the response in the aftermath, and as well as participants' personal and community resources in the aftermath of the hurricanes. Quotes included in this report may come from interviews conducted in English or Spanish, and those from interviews conducted in Spanish were translated for dissemination purposes.

## Results

A total of 16 semistructured interviews were completed. The age range of participants was 26–68 years. Participants of the eight interviews conducted with people living in PR came from different municipalities in the islands, including the San Juan Metropolitan area, as well as the Western, Eastern, Southern, and Central areas. Participants were evenly distributed by sex, and among them were included executive directors of community-based organizations, outreach workers, journalists, and retirees.

Similarly, the eight interviews conducted with Puerto Ricans in the diaspora voiced the experiences of those living in the Northeast, Central, Southern, and Midwest sections of the continental United States. Most participants were women (seven out of eight) and had different experiences, including artists, graduate students, and entrepreneurs. On average, they have been in the continental United States for nearly ten years, ranging from people who moved less than 2 years ago to those who were born in the continental United States. The age and geographical distributions, as well as lived experiences among those in PR and the diaspora, are consistent with diversities in terms of socioeconomic distributions in PR and with the concentrations of different waves of emigrants and generations of Puerto Ricans in the diaspora.

In the analysis conducted, three main themes emerged across narratives as frequent and as illustrative of the issues of race/racism and response to natural disasters in PR. These three themes were “Race, racism, and “fitting the box”,” “Race in emergency and recovery response,” and “Community philanthropy.”

### Race and “fitting the box”

Among those living in PR, the terms most frequently used to self-report their race was “puertorriqueño.” As shared by one of the participants from the Puerto Rican diaspora, even in the presence of options such as Latino or Hispanic, the answer is the same; “You are asking about my race? I am puertorriqueña.”

Among Puerto Ricans in the diaspora, there were other terms used to self-identify his or her race, such as *Latino* or *Hispanic*. As explained by one of the participants, this might be a by-product of having been exposed to “fitting the box” of race. Other participants shared this experience of feeling forced to self-identify with certain racial terms.

Nonetheless, the self-identification with any specific race was often contextualized by participants. As included in Table 1, for those in the diaspora, self-identifying as Latino or Hispanic might be related to a broader sense of community and a mechanism of visibility and empowerment. Participants from PR argued that categories used in the United States for race are contextually meaningless. Overall, participants viewed themselves as a national group with a common history, culture, and heritage distinct from the United States. Race was not perceived as a problem, but experiences of racism were prevalent. As shared by a woman in the diaspora, in PR some of these experiences of racism intersect with other identities identified as minority such as migrant populations.

**Table 1.** Themes Related to Race and Racism in the Aftermath of Natural Disasters from Interviews Conducted with Puerto Ricans in the Archipelago and the Diaspora

Theme	Definition	Example quotes
Race and “fitting the box”	Person's self-identification with one or more social groups in the context of the sociopolitical relationship of Puerto Rico with the United States	“When I was in Puerto Rico I only had to use those terms [Latina or Hispanic] when completing documents that were sent from the US.” As shared by other participant “[…] having to share that I am a Latino is convenient for statistical purposes. I don't know what that [Latino] means in Puerto Rico.”
		“You know, I never used those terms [Latina or Hispanic] when I was living in Puerto Rico, but here in the states gives me a sense that I belong to another group, a minority. I use it more when I am to take a position and to make my minority status visible.”
		“I remember been told by my mother not to shared that I was mixed to avoid racism [in Puerto Rico], but more than racism was to avoid xenophobia, because my father is Dominican. […] However, it was not until I moved to the US [20 years ago] that I learned about racism.”
Racism during emergency and recovery response	Experiences of racism or discrimination due to racial identity in the aftermath of natural disasters	“Puerto Rico has been affected for years of unequal treatment in the context of the United States. Many Puerto Ricans have left the island to find better opportunities. It should not surprise anyone that the country was so weak to face the impact of a major hurricane.”
		Look at how things are in Texas [in reference to the emergency response after hurricane Harvey]. If we were a bunch of whites the response would have been completely different.”
		“Colonialism is not sustainable.”
Community philanthropy	The process of gaining the support, leveraging community resources, and determining the use of external resources in that community to better address challenges	“[We were] prepared with 2 or 3 days of food, but we got really scared after noticing the impact of the devastation and the fact that supermarkets were not open and once open they had limited access or selection.”
		“For two weeks after the hurricane I was able to eat because where I live they were organized to cook one hot meal every day for everybody.”
		“As part of my role in my organization once it was imminent that the hurricane [Maria] was coming I went to the streets to look for my participants to make sure that they had the medication they needed. They could not spend the hurricane without medications. In the process I realized that many were without shelter and unprepared, some pharmacies were already closed, so I used all my networks so they could be ready. At the end, when I came home, I realized that I barely had water and canned food. I was unprepared. I was able to eat for several days after the hurricane because my neighbors cooked and shared with me.”
		“To those with the slogan [#YoNoMeQuito] I hope they can see how we have not quitted. Not been in Puerto Rico does not mean that we cannot do a lot for our island. We are Puerto Ricans wherever we go.”

### Racism during emergency and recovery response

Participants expressed that PR was in the worst circumstances to be prepared for and to recover from a major natural disaster due to the pre-existing socioeconomic and political conditions, including racism, in PR. One participant identified PR's history of “bad governance and corruption” as an aggravating factor to the poor response of the United States. However, participants documented perceived and felt racism during the emergency and the aftermath by comparing how the federal government responded to the natural disaster caused in states such as Texas and Florida. Overall, participants shared that “[…] the response to Hurricane Maria in Puerto Rico is the best evidence of how Puerto Ricans are treated as second-class citizens.”

### Community philanthropy: nuestro granito de arena (our grain of sand)

Endogenous assets are the core component of what we refer to as community philanthropy.^29,30^ As shared by participants, evidence of community philanthropy quickly surfaced just days after the impact of Hurricane Maria in PR.

Participants shared their own experiences or the experiences of their families or communities in the aftermath of the hurricanes in PR. Most participants agreed that many in PR suffered from not having electricity and having limited access to water and communication outlets. However, access to food in the aftermath of the hurricane was a common unexpected problem. Access to food was frequently possible through their neighbors and communities. Sometimes the lack of preparedness was associated with responding to the needs of others in the community in preparation for the hurricane. Nonetheless, the community responses were perceived as organized and described as a “protocol.”

A common comment from the Puerto Ricans in the diaspora was framed by their perceived knowledge of what was going on in PR as portrayed by the US media and their initial perceived inability to do something, but the urge to act; “I couldn't stay home with my arms crossed.” Furthermore, some shared their feelings of frustration and despair; “I should have been there.”

In the past few years, after the significant migration of Puerto Ricans to the continental United States, a social campaign title #*YoNoMeQuito*/#IDoNotQuit has caused mixed responses, and interviewees from the diaspora spontaneously mentioned it. To some, the campaign is just a cover-up of the push to emigrate caused by the socioeconomic crisis, whereas for others it is a motto that reflects their commitment to PR. As shared by one participant, their response evidenced that no matter where the diasporic Puerto Ricans are, they are committed to their country.

## Discussion

Data from this study support the notion that, even when racism is obvious, internalized, and enacted among some Puerto Ricans, PR's political status serves to create a dichotomy whereby the United States is perceived as a highly racialized and racist country relative to Puerto Rican society, where, although racism is prevalent, race is not represented as a problem.^17^ Nonetheless, the racial self-identification as “puertorriqueña” in the context of the United States can be understood as a rejection of US-based racial categories, but may also demonstrate a lack of acknowledgment of racism among people in PR.

Racism is perceived as a barrier to proper response in the aftermath of natural disasters. Participants provided a clear understanding of the inequitable response when compared with states with predominantly white populations and the unsustainability of a colonial status that act as a barrier to federal and international aid (e.g., the impact of the Jones Act). Furthermore, and as previously argued, data from this study support that what PR experienced since September 2017 was a perfect storm caused by major hurricanes and a human-made financial crisis manufactured by bankers and a predatory class of investors.^1^

Structural racism fueled the chaotic disaster aftermath, and community philanthropy should be further explored as a response to the resiliency and resistance reported by Puerto Ricans and as an opportunity to achieve equity. Community philanthropy is inhibited by structural constraints—such as racism—and can be enabled and enhanced by the provision of resources by governments, authorities, and organizations. We can argue how resilience and resistance facilitated community philanthropy as Puerto Ricans were able to have the necessary resources and were capable of organizing before and after the impact of the hurricanes.^31^ In this case, community philanthropy seems to serve as a bridge from PR to the continental United States despite the structural racism evidenced in the PR–US relationship. Nonetheless, our understanding of community philanthropy certainly does not mean that Puerto Ricans or any other community should be left to its own devices in and through policy and planning.

## Conclusion

The colonial status of PR has historically supported a complex relationship with the United States and Puerto Ricans experienced structural racism in the aftermath of natural disasters. Racism was a detrimental determinant for emergency and recovery response in the aftermath of natural disasters in PR. Because there is evidence of community resilience mushrooming from Puerto Rican communities and of community resistance,^1^ the task at hand is to effectively identify and then strengthen the strategies and practices that wittingly or unwittingly will help to reduce health inequities before and in the aftermath of disasters.

## Abbreviations Used

FEMA: Federal Emergency Management Administration
PR: Puerto Rico
